# Morphological predictors of *BRCA1* germline mutations in young women with breast cancer

**DOI:** 10.1038/bjc.2011.41

**Published:** 2011-02-22

**Authors:** M C Southey, S J Ramus, J G Dowty, L D Smith, A A Tesoriero, E E M Wong, G S Dite, M A Jenkins, G B Byrnes, I Winship, K-A Phillips, G G Giles, J L Hopper

**Affiliations:** 1 Department of Pathology, Genetic Epidemiology Laboratory, Victoria, Carlton, Australia; 2 Department of Gynaecological Oncology, UCL EGA Institute for Women's Health, University College London, London, UK; 3 Department of Public Health, Centre for Molecular, Environmental, Genetic and Analytic Epidemiology, School of Population Health, The University of Melbourne, Carlton, Victoria, Australia; 4 The International Agency for Research on Cancer, Lyon, France; 5 Department of Medicine (Royal Melbourne Hospital), The University of Melbourne, Parkville, Victoria, Australia; 6 Department of Genetics, Royal Melbourne Hospital, Parkville, Victoria, Australia; 7 Division of Haematology and Medical Oncology, Peter MacCallum Cancer Centre, Victoria, East Melbourne, Australia; 8 Department of Medicine, St Vincent's Hospital, Victoria, East Melbourne, Australia; 9 Cancer Epidemiology Centre, The Cancer Council Victoria, Victoria, Carlton, Australia

**Keywords:** BRCA1, early-onset breast cancer, tumour morphology

## Abstract

**Background:**

Knowing a young woman with newly diagnosed breast cancer has a germline *BRCA1* mutation informs her clinical management and that of her relatives. We sought an optimal strategy for identifying carriers using family history, breast cancer morphology and hormone receptor status data.

**Methods:**

We studied a population-based sample of 452 Australian women with invasive breast cancer diagnosed before age 40 years for whom we conducted extensive germline mutation testing (29 carried a *BRCA1* mutation) and a systematic pathology review, and collected three-generational family history and tumour ER and PR status. Predictors of mutation status were identified using multiple logistic regression. Areas under receiver operator characteristic (ROC) curves were estimated using five-fold stratified cross-validation.

**Results:**

The probability of being a *BRCA1* mutation carrier increased with number of selected histology features even after adjusting for family history and ER and PR status (*P*<0.0001). From the most parsimonious multivariate model, the odds ratio for being a carrier were: 9.7 (95% confidence interval: 2.6–47.0) for trabecular growth pattern (*P*=0.001); 7.8 (2.7–25.7) for mitotic index over 50 mitoses per 10 high-powered field (*P*=0.0003); and 2.7 (1.3–5.9) for each first-degree relative with breast cancer diagnosed before age 60 years (*P*=0.01).The area under the ROC curve was 0.87 (0.83–0.90).

**Conclusion:**

Pathology review, with attention to a few specific morphological features of invasive breast cancers, can identify almost all *BRCA1* germline mutation carriers among women with early-onset breast cancer without taking into account family history.

About 5–10% of young women diagnosed with breast cancer carry germline mutations in BRCA1 (Newman *et al*, 1998; Southey *et al*, 1999; Malone *et al*, 2000; Ozcelik *et al*, 2003). Knowing a woman's *BRCA1* mutation status soon after the time of diagnosis is important because it could inform her immediate treatment choices, particularly with regard to breast conservation therapy *vs* mastectomy (Pierce *et al*, 2010), and perhaps in the future, her use of targeted therapies, such as Poly (ADP-ribose) polymerase inhibitors (Goodwin *et al*, 2007; Fong *et al*, 2009). Her mutation status is also important for her female relatives because a substantial proportion of these women will carry the same mutation and so will therefore be at high risk of breast and ovarian cancer (Antoniou *et al*, 2003) and will have different preventive and screening options than the general population.

What is the best approach to identify the *BRCA1* mutation carriers among young women with newly diagnosed breast cancer, in terms of timeliness, sensitivity, specificity? Family history is difficult to collect well, especially in a busy clinical setting, in the context of a young woman newly diagnosed with breast cancer. In addition, the woman might not know her family cancer history details and gathering the relevant data and ensuring its accuracy might involve other family members, a process that can be time consuming. Even when accurately reported, well collected and verified cancer family history is often uninformative, unless extreme, as it is neither sensitive nor specific to *BRCA1* mutation status. The areas under the receiver operator characteristic (ROC) curves for algorithms based on family history alone, such as BRCAPRO and BOADICEA, are at most 0.7–0.8 (e.g., Antoniou *et al*, 2008b). The odds of being a carrier increases on average by only a few fold for each first-degree relative with breast cancer (Ozcelik *et al*, 2003; Apicella *et al*, 2007) and given that the mutation frequency in the general population is about 1 in 600 (Antoniou *et al*, 2008a), it can be seen that family history must be extreme before the probability of being a carrier exceeds a nominal and historic testing threshold of 10% (Burke *et al*, 1997), let alone the newer UK benchmark of 20% (National Institute for Clinical Excellence, 2004). Even for the women with apparently strong family histories being tested by cancer genetics services, the great majority (>70%) are found to be non-carriers (Parmigiani *et al*, 2007; Antoniou *et al*, 2008b). Further, large proportions of the women who have been tested have had low *a priori* probability of being a carrier. For example, in a high-risk setting in the United States >40% of those tested were below the 10% threshold based on BRCAPRO (Parmigiani *et al*, 2007), and similar figures have been observed in the United Kingdom (Antoniou *et al*, 2008b).

It has been known for some time that there are particular morphological and histopathological features evident on tumour review that are more common in cancers arising in *BRCA1* mutation carriers (Lakhani *et al*, 1998). These features have been identified by studying carriers across a wide range of ages at diagnosis who were ascertained because of their strong family cancer history. Lack of ER and PR expression have also been reported to improve prediction of *BRCA1* mutation status based on family history (Lakhani *et al*, 2002; James *et al*, 2006; Mavaddat *et al*, 2010). To date, no validated algorithm appears to have been developed based on all of these predictive features, let alone using a population-based series of early-onset breast cancers.

Our aim was to devise a practical strategy that could be applied at the time of diagnosis for identifying, with high sensitivity and specificity, those young women with early-onset breast cancer who have the highest probability of carrying a germline mutation in *BRCA1*. We used morphological and immunohistochemical data that could be routinely collected at diagnosis, as well as data on family history of breast cancer in first- and second-degree relatives.

## Participants and Methods

### Australian Breast Cancer Family Registry

The Australian Breast Cancer Family Registry (ABCFR) includes a population-based, case–control–family study of breast cancer, in which cases and controls (probands) and their relatives were administered the same questionnaires, carried out in Melbourne and Sydney, Australia (Hopper *et al*, 1994, 1999; McCredie *et al*, 1998). It is a component of the international Breast Cancer Family Registry (John *et al*, 2004; Neuhausen *et al*, 2008). The study was approved by the ethics committees of The University of Melbourne and The Cancer Councils of Victoria and New South Wales. All participants provided written informed consent for participation in the study.

### Family history data

For each family, a pedigree was constructed by interviewing the proband and all participating relatives, covering all known first- and second-degree adult relatives of the proband, their vital statuses, cancer histories, dates of birth and, if appropriate, dates of death and/or diagnosis. Consequently, reports of cancer in relatives came from multiple sources within the family, so that the pedigree information for each person was based on a self-report or report(s) from first-degree relatives and rarely was only on a report from a second-degree relative. Attempts were made to verify cancer reports using cancer registries, death certificates and other medical records (Dite *et al*, 2003). For probands, a strong family history was defined as having two or more first- or second-degree relatives diagnosed with breast or ovarian cancer (including male breast cancer) on the same side of the pedigree.

### Tumour retrieval, pathology review and morphology score

We attempted to retrieve archival blocks for the first primary invasive breast tumours of all 856 probands diagnosed before the age of 40 years. A total of 452 tumours (53%) were retrieved and included material from 16 surgical biopsies, 7 spot/needle localisations, 309 lumpectomy/quadrantectomies, 167 mastectomies and 442 axillary dissections. The mean age at diagnosis for these retrieved cases was 35 years, as it was for the 856 probands. Histological type was recorded for 442 (98%) of the reviewed cases, the majority of the tumours were infiltrating ductal carcinomas (NOS) (83%). Atypical medullary (5%), classical medullary (1%), pleomorphic lobular (4%), classical lobular (2%) and other very rare histological types (tubular, cribiform, mucinous, secretory, metaplastic and alveolar lobular, 4%) made up the remainder.

These were scored for morphology features by a pathologist blind to the mutation status as described by Armes *et al* (1998) and Longacre *et al* (2006). Briefly, the tumours were typed into primary pattern and secondary pattern using the World Health Organisation breast carcinoma classification with minor modifications as described by Page *et al* (1987). Tumour grade was scored using the modified system of Bloom and Richardson by assessing mitotic rate, tubular differentiation and nuclear pleomorphism (Page *et al*, 1987).

We then selected nine features that have been reported to be associated with *BRCA1* germline mutation status (Eisinger *et al*, 1996; Breast Cancer Linkage Consortium, 1997; Jóhannsson *et al*, 1997; Armes *et al*, 1998; Lakhani *et al*, 1998):

1. Very high mitotic index defined here (see justification below) as >50 mitoses per 10 high-powered fields (h.p.f.). The mitotic index of each tumour was scored as: number of mitoses per 10 h.p.f.
2. High nuclear grade (score of 3 for nuclear pleomorphism according to Elston (1987), which is scored as: (1) bland, (2) intermediate or (3) malignant (high)).
3. Little or no tubule formation (score of 3 for tubule formation (<10%)) according to Elston (1987), which is scored as: (1) >75%, (2) 10–75% and (3) <10%).
4. Trabecular growth pattern. Primary and secondary growth patterns are scored as: (1) acinar (organoid), (2) lobular, (3) trabecular and (4) tubular. Primary trabecular growth pattern was scored according to Ridolfi criteria for medullary cancers requiring >75% of the tumour to have a trabecular growth pattern (Ridolfi *et al*, 1977).
5. Pushing margin, defined here as a continuous front of cells observed in >50% of the tumour circumference and scored as: (1) yes and (2) no.
6. Circumscribed growth pattern scored as: (1) yes and (2) no.
7. Syncytial growth pattern >75% scored as: (1) yes or (2) no.
8. Necrosis scored as: absent or present; and
9. Moderate or intense lymphocytic infiltrate, scored as: (1) absent/minimal, (2) moderate or (3) intense.

We generated a morphology score for each tumour by adding up the number of the following features that were present; high mitotic index, high nuclear grade, little or no tubule formation, trabecular growth pattern, pushing margin, circumscribed growth pattern, syncytial growth pattern, necrosis and a moderate or intense lymphocytic infiltrate, so that the score ranged from 0 to 9.

ER and PR status had been collected by the ABCFR for 402 (89%) of the 452 reviewed breast cancer cases. This information was collected from the state cancer registries (58%), diagnostic pathology reports (29%) and from immunohistochemical staining of tumour tissues (13% Armes *et al*, 1998) as described in McCredie *et al* (1998).

### *BRCA1* mutation screening

For 788 (92%) of the probands, and for all 455 of the reviewed cases, previous *BRCA1* mutation screening included:

1. Protein truncation testing covering exon 11 of *BRCA1* (Hopper *et al*, 1999).
2. Manual sequencing of the coding and flanking intronic regions of *BRCA1* for: (i) a random sample of 91 probands (Southey *et al*, 1999), (ii) 63 probands with a strong family history of breast and/or ovarian cancer, (iii) 6 probands whose mothers had two primary breast cancers and (iv) 9 probands who had at least two or first- or second-degree relatives (maternal and paternal) with breast or ovarian cancer.
3. Large genomic alteration screening of *BRCA1* using multiplex ligation-dependent probe amplification (MLPA) as previously described (Smith *et al*, 2007) for groups (ii), (iii) and (iv) in (2) above.
4. Testing for the two Ashkenazi founder mutations in *BRCA1*, 185delAG and 5238insC for all (Leong *et al*, 2000; Dite *et al*, 2003; Apicella *et al*, 2007).
5. Testing for the duplication of exon 13 in *BRCA1* for 641 probands as described previously (Puget *et al*, 1999; Leong *et al*, 2000).

Definition of pathogenicity for *BRCA1* sequence variants was consistent with the policy of the Breast Cancer Information Core database (http://research.nhgri.nih.gov/bic/) and Neuhausen *et al* (2008). The above testing identified 39 pathogenic mutation carriers. The breast cancers arising in 29 (74%) of these carriers were retrieved and reviewed.

### Statistical analysis

Associations between features were assessed by dichotomising and calculating corresponding odds ratios (ORs). Associations between the outcome, mutation status and potential predictors were estimated using simple and multiple logistic regression. The best fitting model was defined to be the one with the lowest Bayesian information criterion (BIC) (Schwarz, 1978) using observations with no missing data for any variable (*N*=400). *P*-values were based on the likelihood ratio test unless otherwise indicated and all calculations were performed using R version 2.7.2 (R Development Core Team, 2010).

The performance of the best fitting model was assessed using the same data set on which the model was fitted using a five-fold stratified cross-validation approach, as recommended by Kohavi (1995).

The predicted probability that a woman carries a BRCA1 mutation according to the best fitting model was calculated in the standard way as f(*x*^T^*β*) where f is the logistic function given by f(*L*)=exp(L)/(1+exp(L)), *β* is the column matrix of maximum likelihood estimates, and *x* is the relevant part of the woman's column matrix of covariates. Asymptotic likelihood theory implies that *β* is normally distributed and allows estimation of its variance–covariance matrix Σ so it follows that *x*^T^*β* is normally distributed with variance *σ*^2^=*x*^T^Σ*x*. A 95% confidence interval (CI) for the predicted carrier probability, f(*x*^T^*β*), was obtained by applying f to the limits, *x*^T^*β*±1.96*σ*, for the predicted log-odds.

## Results

**Figure 1:**
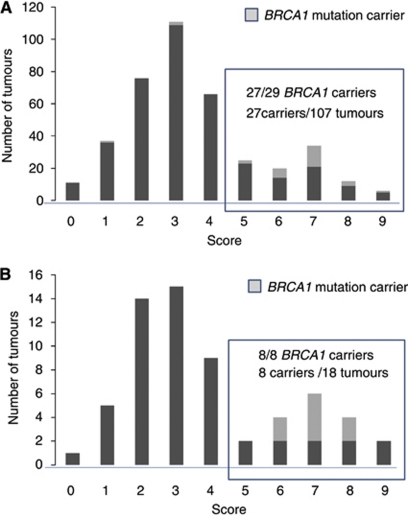
Morphology scores of all early-onset breast cancer (**A**) and early-onset breast cancer with a strong family history (**B**).

A total of 29 (6%) of the 452 probands whose tumours had undergone pathology review were found to be *BRCA1* mutation carriers. The odds of being a carrier depended strongly on the morphology score, increasing on average by 80% (95% CI: 40–240%) with each additional feature after adjusting for family history and ER and PR status (*P* for trend <0.0001). Figure 1A shows that mutation carriers tended to have higher morphology scores than non-carriers; of the carriers, 27 (93%) had a morphology score of 5 or more compared with 25% of all probands. Figure 1B shows that, when restricted to probands with a strong family history, the distribution of the morphology score was bimodal. All 10 carriers with a strong family history also had a morphology score of 5 or more, and these carriers comprised 48% of all probands with a strong family history and a morphology score of 5 or more.

Table 1 shows that some characteristics of family history were associated with mutations status, but no family history feature alone was highly sensitive (maximum 41%) and all had low positive predictive values (<0.25%).

Table 1 also shows that, in contrast to family history features, most morphological features studied were individually predictive of *BRCA1* mutation status, with four having smaller *P*-values than even the most significant family history feature. In general, the morphological features had high sensitivities (all but three>75%) and specificities (all but three⩾65%). Moderate or intense lymphocytic infiltrate, nuclear grade and little or no tubule formation had high sensitivities but low specificities while syncytial growth pattern, pushing margins and circumscribed growth pattern had high specificities but low sensitivities. The predictive features with crude OR>10 were: trabecular growth pattern, high mitotic index, and necrosis (sensitivity⩾79%, specificity⩾65%, positive predictive value 0.14–0.32).

Of the 452 tumours reviewed, 154 had mitotic indices between 0 and 9/10 h.p.f. and of these 1 (1%) was a *BRCA1* mutation carrier, 115 had mitotic indices between 10 and 19/10 h.p.f and of these 1 (1%) was a *BRCA1* mutation carrier, 111 had indices between 20 and 49/10 h.p.f. and of these 2 (2%) were *BRCA1* mutation carriers and 72 had mitotic indices of 50 or more/10 h.p.f. (range 0–292/10 h.p.f.) and of these 25 (35%) were *BRCA1* mutation carriers. A total of 74 tumours were scored to have primary trabecular growth pattern and 36 were scored to have a secondary trabecular growth pattern (all these 36 tumours were scored to have a primary acinar growth pattern).

ER and PR statuses were both individually predictive of mutation status, but while they had high sensitivities they did not have high specificities and alone their positive predictive values were each only 0.13. Being negative for both ER and PR was associated with an OR of 5.3 (95% CI: 2.3–13.0), sensitivity of 70%, specificity of 67% and positive and negative predictive values of 13 and 97%, respectively.

**Figure 2:**
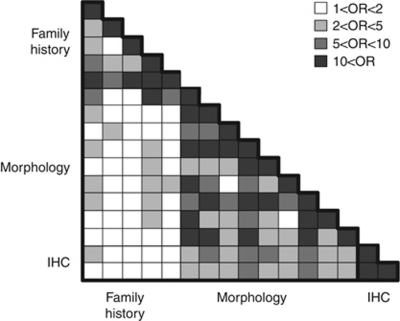
Levels of association between the features listed in Table 1. Each row and column corresponds to a feature (in the same order as in Table 1) and the shading represents different levels of odds ratios (ORs), as indicated in the figure. Odds ratios that were <1 were shaded the same as their reciprocals.

Figure 2 shows that the features within each of the three categories (family history, morphology and immunohistochemistry) were strongly associated with each other. The morphology features and ER and PR status were generally associated with each other but not with the family history features.

When models containing multiple features were considered, the best fitting model (as judged by BIC) included just three features. These features and their jointly estimated ORs (95% CI, Wald *P*-value) were: trabecular growth pattern, OR=9.7 (2.6–47.0; *P*=0.001); high mitotic index, OR=7.8 (2.7–25.7; *P*=0.0003); and number of first-degree relatives with breast cancer diagnosed before the age of 60 years, OR for each relative=2.7 (1.3–5.9; *P*=0.01). The area under the ROC curve for this model was 0.87 (95% CI: 0.83 to 0.90). The OR estimates for the two morphology features were negatively correlated with each other (*r*=−0.44) but neither was correlated with the OR estimate for family history (*r*=−0.04 and 0.09).

Table 2 shows cross-validation estimates of the areas under the ROC curves for models that included the most predictive features from one or more of the three categories of morphology, immunohistochemistry (comprising ER and PR receptor status) and family history. It is apparent from Table 2 that the morphology features have the greatest impact on the area under the ROC curve, and that models that include the morphology features are not improved by the addition of family history variables or ER and PR status.

Under the best fitting model, the probability that a woman diagnosed with breast cancer before age 40 years carries a *BRCA1* mutation is exp(*L*)/[1+exp(*L*)] where the log-odds *L* is given by the formula *L*=−5.1544+2.0539 *x*+2.2750 *y*+0.9784 *z* and: *x* is 1 if the woman's tumour has high mitotic index and 0 otherwise; *y* is 1 if the woman's tumour has a trabecular growth pattern and 0 otherwise; and *z* is the number of the woman's first-degree relatives who have been diagnosed with breast cancer before the age of 60 years. A list of predicted probabilities for various values of *x*, *y* and *z* are given in Table 3.

The fit of the best model was not improved by more than could be attributed to chance by the addition of either ER and/or PR receptor status. After adjusting for the three variables in the best fitting model, the ORs (95% CI) associated with negative ER and PR receptor statuses were 1.8 (0.5–7.1) and 1.5 (0.6–4.4), respectively (both *P*=0.3).

Of the 58 probands whose tumours had both high mitotic index and trabecular growth pattern, 21 (36%) were *BRCA1* mutation carriers. Of the 332 probands who had neither of these morphological features, only one (0.3%) was a carrier. Of the 123 who had one or both features, 28 were carriers, and this was associated with OR=98 (95% CI: 20–1750), sensitivity=97%, specificity=78% and positive predictive value=23%.

Of the 156 (34%) probands who had one or more of the three features high mitotic index, trabecular growth pattern and one or more first-degree relatives diagnosed with breast cancer before the age of 60 years, 28 (18%) were carriers. Of the 70 probands who had two or more of these three features, 24 (34%) were carriers.

## Discussion

Our study has shown that, by considering just two tumour morphological features that could potentially be reported at the time of diagnostic pathology, and one aspect of family history of breast cancer, it is feasible to establish a simple way to identify those young women with breast cancer who are most likely to carry a *BRCA1* germline mutation. Even if not supplemented by the information on family history, a trabecular growth pattern and a high mitotic index high were strong indicators of a woman's *BRCA1* mutation status. Moreover, knowing ER and PR status did not improve the model predictions once the two morphology features and a family history variable were taken into account. In the context of breast cancers arising in young women, we found that any *BRCA1* predictive value of these two measures appears to be subsumed by the two key morphological features. It would be of interest to know if this applied to breast cancers in women with later age at diagnosis.

For our population-based sample of women with early-onset breast cancer, if *BRCA1* screening had been restricted to those with a strong family history then we would have screened 71 cases and found 10 carriers (14%). If instead we had screened those with a high mitotic index, we would still have screened 71 cases but found 23 carriers (32%), more than twice as many. If we also screened those with a trabecular growth pattern, we would have screened a further 53 cases and found another 5 carriers (10%). Therefore, screening those with high mitotic index and/or trabecular growth pattern would have found 28 *BRCA1* mutation carriers in 124 women screened (24%) and missed only one carrier. Screening only one-quarter of early-onset cases based on just these two morphology features would have been sufficient to identify almost all carriers, without any reference to family history.

Table 2 shows the area under the ROC curve (AUC), an omnibus measure of a model's predictive power. Knowing family history in addition to the morphology features made no improvement in the AUC. The AUC based on family history alone was less than that based on ER and PR, and when used together was still less than the AUC based on the two key morphology features alone. In terms of predictive strength, the traditional indicators – measures of family history – were weaker than most of the morphological features. Our family history data are likely to be no less accurate and complete than family history data collected in a clinical setting.

This work has also identified a fascinating group of early-onset breast cancers that share the morphological features of early-onset breast cancers that carry identifiable *BRCA1* mutations. These are the cancers, illustrated in Figure 1A, that have a morphology score of 5 or more but, despite extensive testing, are from women for whom we have not been able to identify germline *BRCA1* mutations. We performed further *BRCA1* mutation screening on DNA samples from the women whose tumours had mitotic indices over 50 per 10 h.p.f. and a trabecular growth pattern and who were not already known to carry a mutation in *BRCA1* (*n*=37). (The mutation testing that had been performed on DNA from these women was varied because of the previous mutation testing strategy described above in the methods). A further two *BRCA1* mutations, *BRCA1* del1A-23 and *BRCA1* delexon20, were identified by MLPA testing. The woman with the *BRCA1* del1A-23 mutation had breast cancer diagnosed at the age of 33 years and a paternal grandmother with breast cancer diagnosed at age 71 years (verified). The women with the *BRCA1* delexon20 mutation had a breast cancer diagnosed at the age of 31 at recruitment, and subsequently another at age 35 years, and had an unverified report of breast cancer in a paternal grandmother (age at diagnosis unknown).

Using tumours from multiple-case breast cancer families with on average later age at onset than in our series, Lakhani *et al* (1998 and 2002) carried out similar studies to ours to try to identify *BRCA1* mutation carriers on the basis of pathology and immunohistochemistry. The ORs for prediction they found were somewhat lower than we observed for early-onset cases. This might be due to a potential change in morphological features associated with *BRCA1* germline mutations in postmenopausal women that could be due to non-germline factors, such as oestrogens and methylation (both known to have a changing role with increasing age).

From our data and model fit, we have calculated the predicted probability of carrying a germline BRCA1 mutation based on the two morphology features and family history: see Table 3. It can be seen that 15% of our sample had predicted probabilities >10% (68 women in 7 categories). On the other hand, for the group of women who had none of the three predictive features, comprising 66% of our sample (299 women), the predicted probability was only 1%. This information could be clinically useful in helping decide where limited resources for counselling and mutation testing for *BRCA1* carriers in women with early-onset breast cancer might best be directed.

Therefore, our data suggest that is possible to institute a pathology-based and more sensitive and specific method for prioritising women with early-onset breast cancer for *BRCA1* mutation testing, similar to that which already applies to colorectal cancer and the DNA mismatch repair genes (Boland *et al*, 1998; Vasen *et al*, 1999; Southey *et al*, 2005; Lenz, 2005; Mead *et al*, 2007). We intend to undertake an independent study to further improve our algorithms, and we encourage others to try to validate and extend this approach, especially to later-onset disease. Pathology review, with attention to a few specific morphological features of invasive breast cancers, can identify almost all *BRCA1* germline mutation carriers among women with early-onset breast cancer without taking into account family history.

## Figures and Tables

**Table 1:** For each feature potentially predictive of *BRCA1* mutation status, the number (frequency in brackets) overall and by *BRCA1* mutation status, the OR with 95% CI and corresponding statistical significance (*P*), and the NPV, PPV, sensitivity (proportion of carriers with the feature) and specificity (proportion of non-carriers without the feature)

**Feature**	**BRCA1 carriers (sensitivity)**	**BRCA1 non-carriers (1-specificity)**	**OR (95% CI)**	***P***	**NPV**	**PPV**
*Family history*	N=*29*	N=*426*				
One or more first-degree relatives with breast cancer <60 years	12 (41%)	48 (11%)	5.6 (2.5–12)	0.0001	0.96	0.2
One or more first- or second-degree relatives with ovarian cancer	2 (7%)	6 (1%)	5.2 (0.74–24)	0.09	0.94	0.25
Strong family history	10 (34%)	61 (14%)	3.1 (1.3–7)	0.009	0.95	0.14
One or more first-degree relatives with breast cancer ⩾60 years	2 (7%)	19 (4%)	1.6 (0.24–5.9)	0.6	0.94	0.1
One or more second-degree relatives with breast cancer	9 (31%)	126 (30%)	1.1 (0.45–2.4)	0.9	0.94	0.07
						
*Morphological features*	N=*29*	N=*426*				
Trabecular growth pattern	26 (90%)	84 (20%)	35 (12–150)	<0.0001	0.99	0.24
High mitotic index	23 (79%)	48 (11%)	30 (12–85)	<0.0001	0.98	0.32
Necrosis	25 (86%)	148 (35%)	12 (4.5–40)	<0.0001	0.99	0.14
Circumscribed growth pattern	18 (62%)	75 (18%)	7.7 (3.5–17)	<0.0001	0.97	0.19
Moderate or intense lymphocytic infiltrate	28 (97%)	339 (80%)	7.2 (1.5–130)	0.008	0.99	0.08
Syncytial growth pattern	8 (28%)	28 (7%)	5.4 (2.1–13)	0.0009	0.95	0.22
Malignant nuclear grade	28 (97%)	361 (85%)	5 (1–91)	0.04	0.98	0.07
Pushing margins (> 50%)	2 (7%)	14 (3%)	2.2 (0.33–8.3)	0.4	0.94	0.12
Little or no tubule formation	25 (86%)	319 (75%)	2.1 (0.79–7.2)	0.1	0.96	0.07
						
*ER and PR status*	N=*27*	N=*375*				
ER negative	23 (85%)	153 (41%)	8.3 (3.1–29)	<0.0001	0.98	0.13
PR negative	19 (70%)	125 (33%)	4.7 (2.1–12)	0.0002	0.97	0.13

Abbreviations: CI=confidence interval; ER=estrogen receptor; NPV= negative predictive value; OR=odds ratio; PPV=positive predictive value; PR=progesterone receptor.

**Table 2:** Cross-validation estimates of areas under the ROC curves from logistic regression models whose predictors were restricted to one or more of the categories of explanatory features given in the table 1

	**Without morphology**	**With morphology**
No family history or ER or PR status	—	0.88 (0.85–0.91)
Family history alone	0.65 (0.61–0.70)	0.87 (0.83–0.90)
ER and PR receptor status alone	0.73 (0.68–0.77)	0.88 (0.85–0.91)
Family history and ER and PR status	0.76 (0.71–0.79)	0.87 (0.83–0.90)

Abbreviations: ER=estrogen receptor; PR=progesterone receptor; ROC=receiver operating characteristic.

**Table 3:** The predicted probability that a woman carries a *BRCA1* mutation according to the best fitting logistic regression model

**Trabecular growth pattern**	**High mitotic index**	**Number of first-degree relatives diagnosed with breast cancer before age 60 years**	***n* (%)**	**Predicted percent probability of carrying a BRCA1 mutation (95% CI)**
Absent	Absent	0	299 (65.7%)	1% (0–2%)
		1	29 (6.4%)	2% (0–5%)
		2	4 (0.9%)	4% (1–18%)
	Present	0	11 (2.4%)	4% (1–16%)
		1	2 (0.4%)	11% (2–36%)
		2	0 (0%)	24% (4–69%)
Present	Absent	0	42 (9.2%)	5% (2–14%)
		1	9 (2%)	13% (5–31%)
		2	1 (0.2%)	28% (8–65%)
	Present	0	43 (9.5%)	30% (19–44%)
		1	11 (2.4%)	54% (36–71%)
		2	2 (0.4%)	76% (44–92%)

Abbreviation: CI=confidence interval.
